# NET: a new framework for the vectorization and examination of network data

**DOI:** 10.1186/s13029-017-0064-3

**Published:** 2017-02-08

**Authors:** Jana Lasser, Eleni Katifori

**Affiliations:** 1 Max Planck Institute for Dynamics and Self-Organization, Göttingen, Am Fassberg 17, Göttingen, 37077 Germany; 2 Department of Physics & Astronomy, University of Pennsylvania, 209 South 33rd Street, Philadelphia, 19104-6396 PA USA

**Keywords:** Network extraction, Data acquisition, Software, Leaf venation, Drosophila

## Abstract

**Background:**

The analysis of complex networks both in general and in particular as pertaining to real biological systems has been the focus of intense scientific attention in the past and present. In this paper we introduce two tools that provide fast and efficient means for the processing and quantification of biological networks like *Drosophila* tracheoles or leaf venation patterns: the Network Extraction Tool (*NET*) to extract data and the Graph-edit-GUI (*GeGUI*) to visualize and modify networks.

**Results:**

NET is especially designed for high-throughput semi-automated analysis of biological datasets containing digital images of networks. The framework starts with the segmentation of the image and then proceeds to vectorization using methodologies from optical character recognition. After a series of steps to clean and improve the quality of the extracted data the framework produces a graph in which the network is represented only by its nodes and neighborhood-relations. The final output contains information about the adjacency matrix of the graph, the width of the edges and the positions of the nodes in space. *NET* also provides tools for statistical analysis of the network properties, such as the number of nodes or total network length. Other, more complex metrics can be calculated by importing the vectorized network to specialized network analysis packages.

*GeGUI* is designed to facilitate manual correction of non-planar networks as these may contain artifacts or spurious junctions due to branches crossing each other. It is tailored for but not limited to the processing of networks from microscopy images of *Drosophila* tracheoles.

**Conclusion:**

The networks extracted by *NET* closely approximate the network depicted in the original image. *NET* is fast, yields reproducible results and is able to capture the full geometry of the network, including curved branches. Additionally *GeGUI* allows easy handling and visualization of the networks.

**Electronic supplementary material:**

The online version of this article (doi:10.1186/s13029-017-0064-3) contains supplementary material, which is available to authorized users.

## Background

The analysis of complex networks both in general and in particular as pertaining to real biological systems has been the focus of intense scientific attention in the past and present [1–3]. However, before a network can be analyzed it has to be imaged and its structure has to be distilled in such a way that it is readable by a computer. This is especially important in times were datasets get increasingly large and manual processing and measurement of quantities like network length or number of branches it not feasible anymore. In the past, several protocols were published which include software and instructions for the segmentation of images and extraction of network data, with special focus on physarum [1, 4], leaves [2, 5] or the animal vasculature [6, 7]. Some of these approaches have been criticized for methodological errors [5, 8] or work as toolboxes of proprietary software [7], therefore limiting their applicability. *NET* and *GeGUI* provide an alternative software solution with an approach that focuses on speed, open access, degree of automation and versatility. One tool that warrants special mentioning is *NEFI* - Network Extraction From Images [9]. It has been developed recently and has strong similarities to *NET* with regards to its open source character and focus on high throughput. The main difference of the two is *NEFI*’s inability to capture the detailed geometry of the network: edges with curves or kinks will be contracted to straight lines. Moreover *NEFI* depends on thinning for its network extraction whereas *NET* follows a vectorization approach which, in our opinion, increases stability and reduces occurrence of artifacts. On the other hand *NEFI* offers strong and versatile tools for image preprocessing and segmentation which are definitely worth considering when extracting networks from noisy images. Like *NEFI*, *NET* is freely available from GitHub [10] and we encourage the reader to download the software and follow the examples shown in this publication.

In general, the problem of extracting a network from an image can be divided into two non-trivial steps:

- Segmentation into foreground and background.
- Vectorization and extraction of network data.

The output of the protocol to generate a well-segmented binary image is highly dependent on the quality and characteristics of the original raw image. Therefore we will only briefly touch upon this subject here and only explain the technique we used to segment the images used in this publication to demonstrate the functionality of *NET*. We have to emphasize that *NET*’s main purpose and also its main strengths lie *after* the image segmentation. The script for performing the segmentation in the repository involves standard image processing methods like adaptive thresholding and is not very sophisticated nor does it fit every dataset. For datasets with heavy noise, intensity gradients or incomplete networks, this script is going to fail. If the images cannot successfully be processed with the script we provide, we recommend looking into more sophisticated tools like *ilastik* [11] or *fiji* [12] or implement and tweak one of the more recent image segmentation methods like the GrabCut [13] or CoopCut [14].

**Fig. 1:**
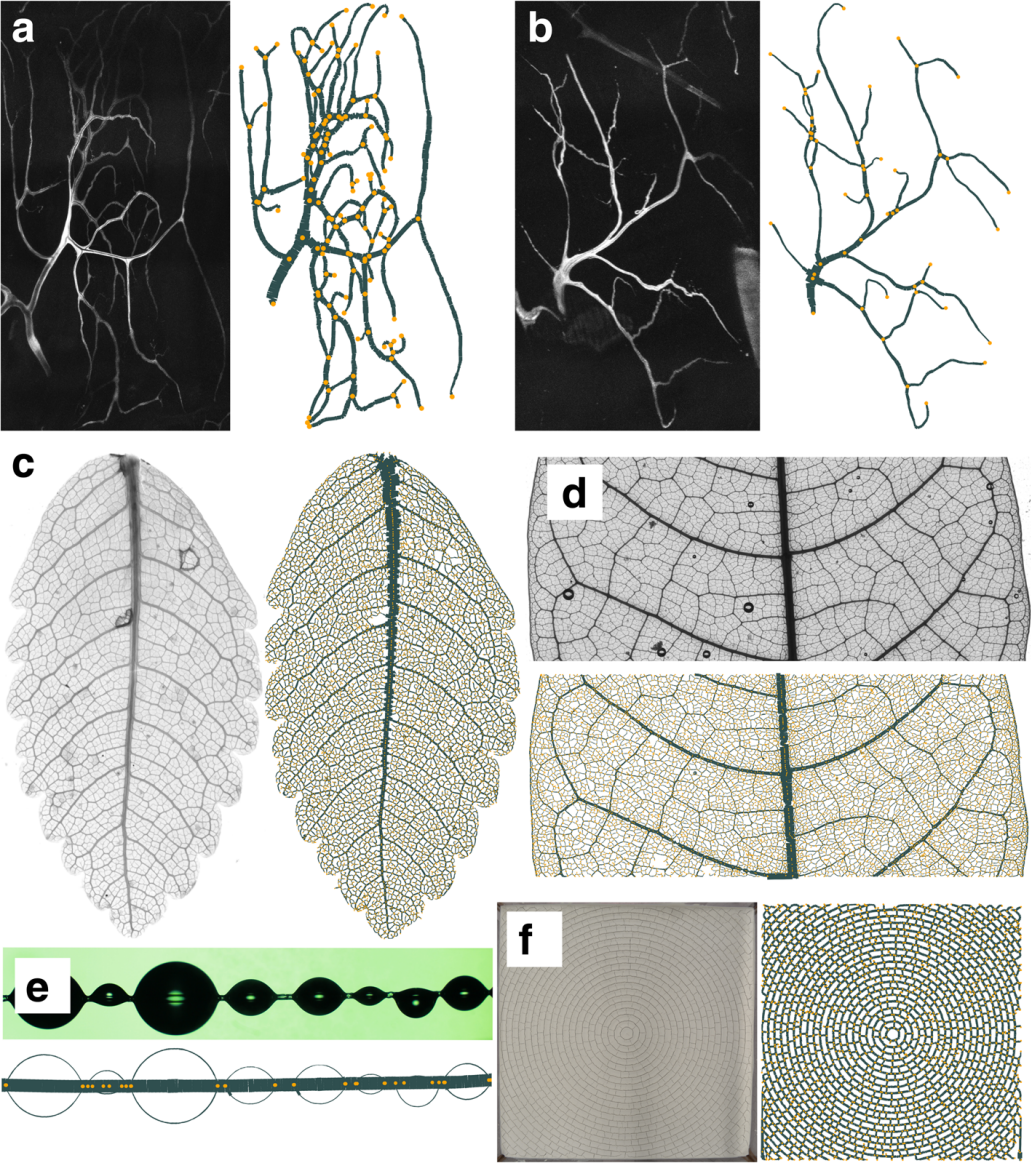
Unprocessed images and vectorized network. The figure names used here refer to the file names of the images in the online repository. **a** and **b** Networks extracted from images *tracheole3* and *tracheole4*. Unprocessed images courtesy of Sara Sigurbjörnsdottir, Leptin Lab, EMBL Heidelberg. **c** and **d** Leaf venation patterns extracted from image *leaf2* and *leaf3*. Unprocessed image courtesy of Douglas Daly, New York Botanical Garden **e** Droplets on a fiber extracted from image *bubbles1*. Unprocessed image courtesy of Marcin Makowski, MPIDS, Göttingen. **f** Crack pattern in clay extracted from image *cracks2*. Unprocessed image courtesy of Pawan Nandakishore, MPIDS, Göttingen. For *leaf3* only a detail is shown because the leaf as a whole is too large for plotting. The images show how *NET* is able to extract the network’s geometry including vertex coordinates and edge trajectories in great detail. Vertices and endpoints of the networks, as indicated by *yellow dots*, as well as edge radii are extracted by the tool

The extraction of network data involves the creation of a skeleton of the shapes present in the binary image and extraction of a graph from the skeleton. This step can easily be generalized as the starting point - the binary images - all have the same basic characteristics. Previous approaches predominately used a pixel based technique called *thinning* [15, 16] to create a skeleton. In this work we choose a different path and create the skeleton following a vectorization approach. The methodology *NET* implements is very stable to noisy features, fast, completely automated and open source and creates graphs that can be easily handled and analyzed. It has already been used for the extraction of network data from *Drosophila* tracheoles as shown in Fig. 1 a, b [17, 18], leaf venation patterns as shown in Fig. 1 c, d [19]. It can be used to track droplets in microfluidics experiments, like in Fig. 1 e and to identify and quantify crack patterns Fig. 1 f [20].

As we want to present a method potentially valuable for other research, the main part of this publication is a description of the computational framework. We describe the workflow enriched with usage examples and give some technical details where they are important for the output of the software. The overview over the framework is followed by a validation of the results generated by *NET*.

## Implementation

**Fig. 2:**
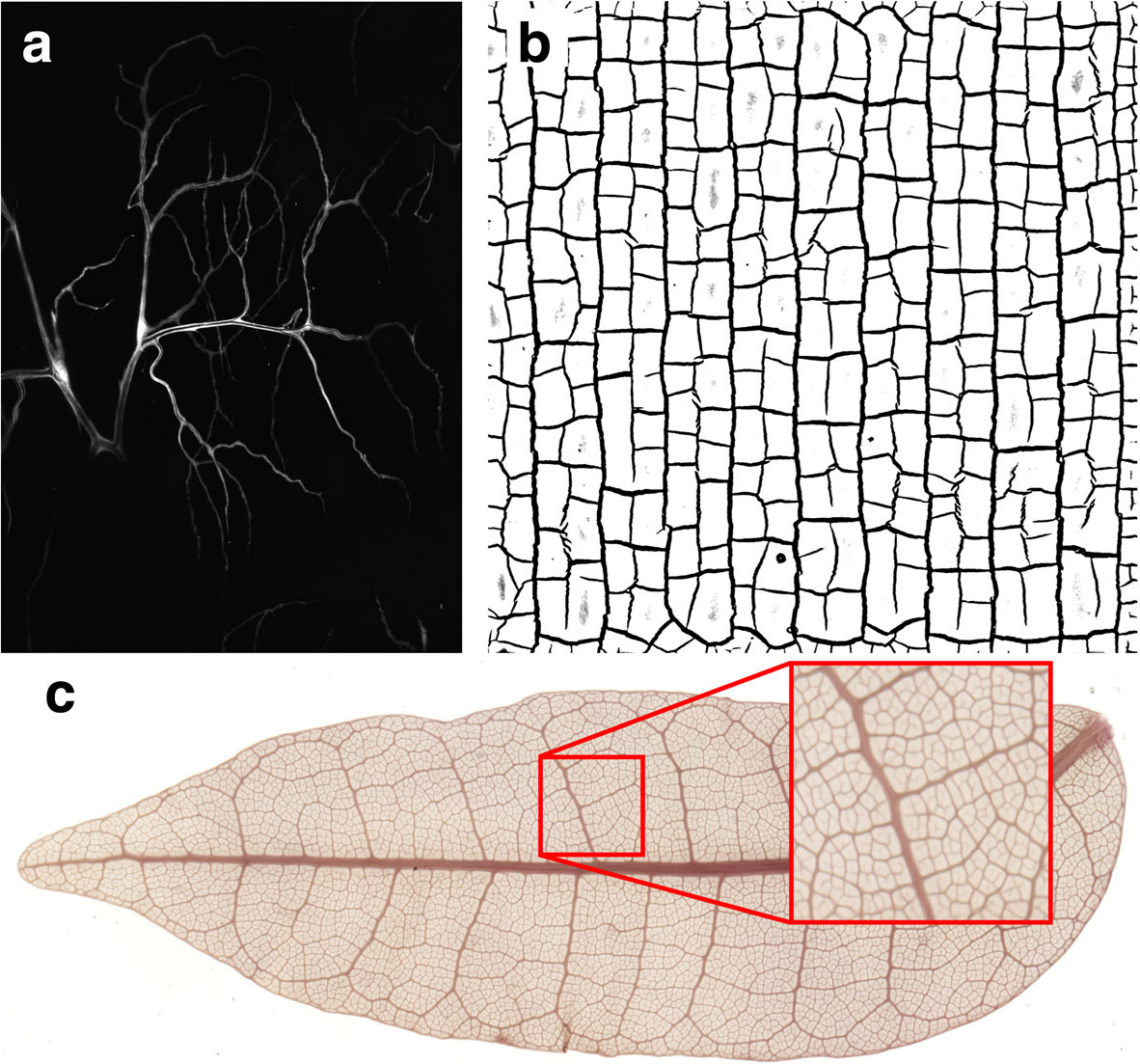
Raw images of natural networks. The corresponding files can be found in the data/originals folder of the repository [10]. **a** Grayscale microscopy image of the branching structure a *Drosophila* Tracheole. Image courtesy of Sara Sigurbjörnsdottir. **b** Digital photograph of cracks in dried clay. Image courtesy of Pawan Nandakishore. **c** High resolution scan (6400 dpi) and zoomed in detail of the vascular network of a *Protium grandifolium* leaf. Cleared leaf sample courtesy of Douglas Daly

The following provides an overview of the functionality of *NET* and *GeGUI* demonstrated on the examples of a *Drosophila* tracheole, a cracked clay surface and a cleared leaf, shown in Fig. 2 a to c. A more thorough description of the technical details is given in the Additional file 1.

The processing steps are carried out by four specialized scripts written in python. Both the scripts and the images used in this publication can be found in the Git repository [10]. We strongly encourage the reader to browse *NET*’s online repository as it is meant to be an integral part of this publication. The publication is written in a way that all results are reproducible by the reader and we give usage examples for every processing step. Before running *NET*, the user will have to install python 2.7 as well as a number of third party libraries on their system. Compatibility with previous versions of python, such as 2.5 and 2.6 is likely but not guaranteed. Some of the code needs to be compiled for the specific platform. *NET* has been designed to work for Linux, Windows and Mac operating systems. Detailed instructions on how to set up the framework on each platform can be found in the readme-file of the repository.

The workflow can be broken down into four main parts:

1. Creating a binary image using either the segmentation script
binarize_adaptive.py or a custom tool or method.
2. Extracting the graph from the network using net.py.
3. Optional: manipulating and manually correcting the graph using gegui.py.
4. Extracting network statistics from the graph using analyze.py.

All scripts are run via the command line. The user needs to provide the path to the file to be processed as required argument. Parameters to modify the script’s behavior are optional:



The repository is organized into one folder for each processing step, containing the necessary scripts. Additionally there is a folder called data/originals containing all the example images used in this publication. All processing steps described in the following paragraphs can be easily reproduced by applying the aforementioned scripts to the images uploaded in the repository at the data/originals folder of [10].

### Step 1: image segmentation

**Fig. 3:**
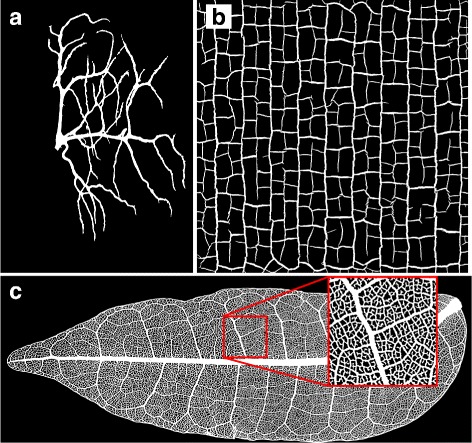
Segmented images of natural networks. Figure names refer to file names in the repository. **a** Binary version of the *tracheole1* image. The left subtree has been digitally cropped. **b** Binary version of the *cracks1* image. No manual editing has been performed. **c** Binary version of the leaf vascular network from image *leaf1*. Small spurious holes in the main vein have been filled

In this step we create a binary representation from the original digital grayscale or color image that contains the network to be analyzed. The goal is to be left with the network as the largest connected structure in the image. Artifacts, stains or noise do not matter as long as they are not connected to the network. How to process the image before a suitable binary image can be created is largely dependent on the characteristics (contrast, definition, resolution) of the image. For some high quality images it might be sufficient to just use thresholding [21] with a constant threshold to separate the network from the background. For most images though it is necessary to employ more sophisticated image processing methods before the thresholding can yield acceptable results. We will not cover these methods here as they are described in depth elsewhere [22]. We provide the basic script we used to create binary images of the examples shown in this publication. These examples all originate in datasets that have been used in real-world scientific projects, the results of which are published for example at [19] and [20]. The script used to segment the images is the same for all the images - only the parameters have been tweaked. The only exception to this is the bubbles-image as it involves edge detection rather than segmentation which is not shown here. The parameters to create each binary image can be found in a text file at the location of the segmentation script. A detailed description of the processing parameters and their impact on the outcome of the binarization process can be found in the Additional file 1. For example to binarize the images of the leaves the user can run:



The resulting binary images are shown in Fig. 3 a to c. For our example images some manual removal of artifacts or cropping needed to be done: In the binary of the tracheole we cropped away the small part of another cell not belonging to the focal tracheole visible in the left of the image. For the leaf we filled in small holes in the main vein. The binary of the crack pattern was not altered. If manual processing steps are involved, the dataset is not suited for automated high throughput processing. Nevertheless if the number of artifacts is negligible, the manual processing steps are not necessary to get sufficiently accurate networks.

Within our segmentation script we provide basic image processing methods to improve the quality of the segmentation. Concerning images that are known to contain long and thin shapes like networks, methods exist to artificially fill in gaps in the network [6, 7]. *NET* does not implement these methods because they might create spurious links and the fidelity of the vectorized network might be hard to validate after application of such a method. This is because a heuristic method such as gap-filling might yield drastically different results on different types of networks or even in different parts of the same network. Such gap-filling algorithms can be incorporated in future versions of *NET*.

### Step 2: extracting the network

In this step we extract the network information from a binary image containing the network structure and distill this information into a weighted planar graph. For this purpose we use the main engine of *NET*, a fast vectorization-based script which creates a very accurate and easy to handle representation of the network. The resulting graph contains information about the spatial position of the nodes and the length and radius of the edges.

**Fig. 4:**
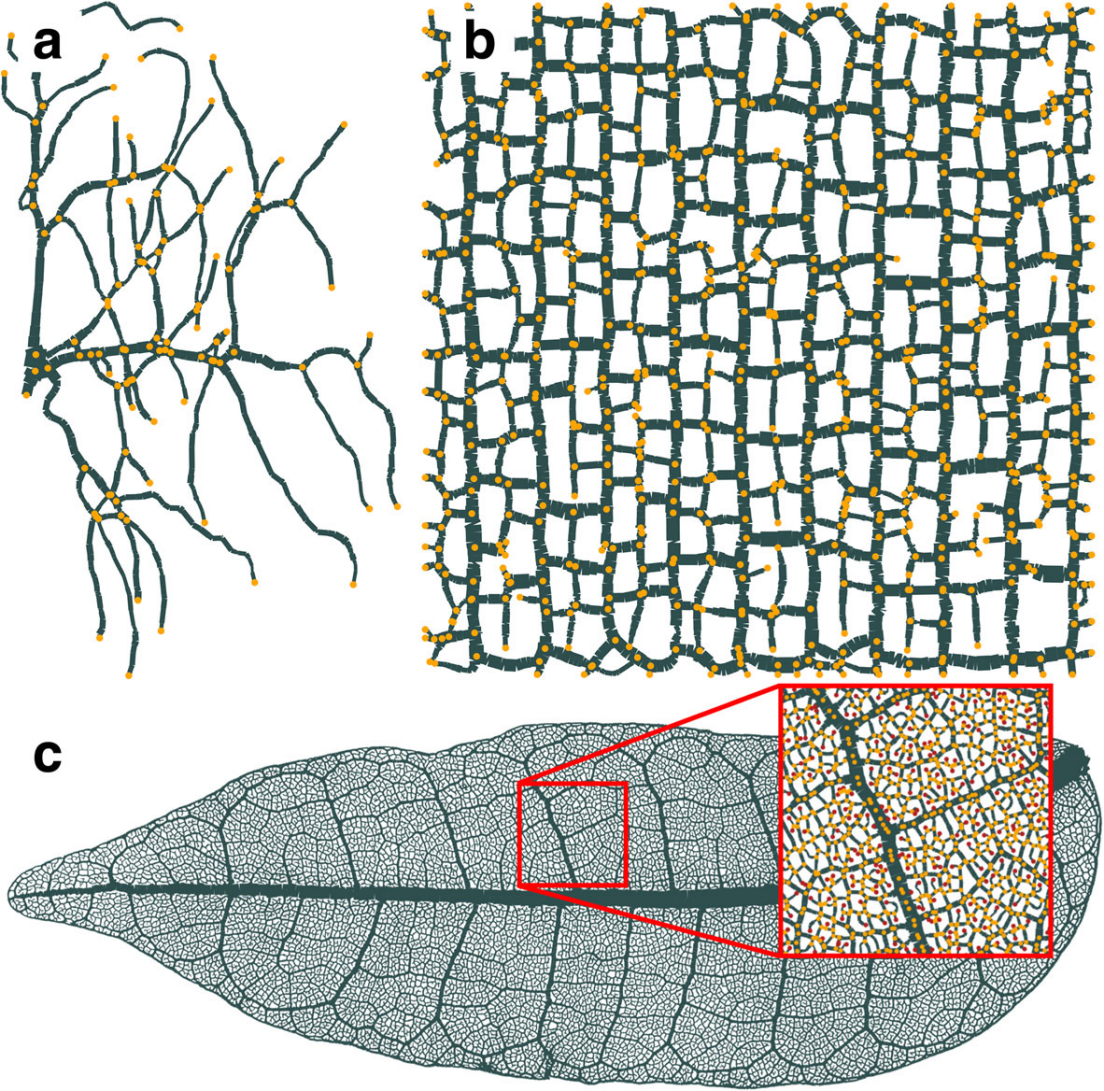
Graphs extracted by *NET* from segmented images of natural networks. The original datasets and resulting graphs are not only different with respect to their geometric layout but also have vastly different sizes. The *yellow points* indicate nodes of the graph whereas *gray lines* represent edges. **a** Graph of the tracheole network. **b** Graph of the crack pattern. **c** Graph of the leaf vascular network

*NET* provides a set of options to modify its behavior depending on the type of graph the user is processing. Most of the times images from the same dataset do not require individual tuning of the script’s options. After the options have been modified to fit one image of the dataset, the script is able to process all the other images with the same options. The most basic use-case of the script requires only the path to the binary image as input. The most important options will be described in this paragraph while a complete list of options is given in the Additional file 1. Figure 4 a to c show the graphs extracted from the binary images in Fig. 3.

To process for example the leaf vascular network *leaf1* from image to graph, the user can run:



For the tracheole network and the crack network we used the parameters *p*=5, *r*=1, *plt*=True and *p*=5, *r*=1, *plt*=True, *fformat*=png, *dpi*=2000 respectively. In the following we will describe the most important parameters to modify the behavior of the script and illustrate the impact of these options on the resulting graph.

**Pruning** **-p** Even with a relatively smooth binary image small kinks in the contour might lead to the emergence of surplus branches in the extracted network. These surplus branches tend to be very short and therefore can be dealt with by pruning away branches shorter than a certain length. Enabling the pruning option removes dangling branches that are shorter than the pruning threshold *p*. Here *p* is not a fixed length in pixels but rather the number of triangles in the triangulation representing the shape. Therefore branches that are shorter than *p* triangles will be removed whereas all other branches remain untouched. Thinning-based algorithms either also prune away smaller branches [23] or use feature characteristics to detect and remove them [24].

**Fig. 5:**
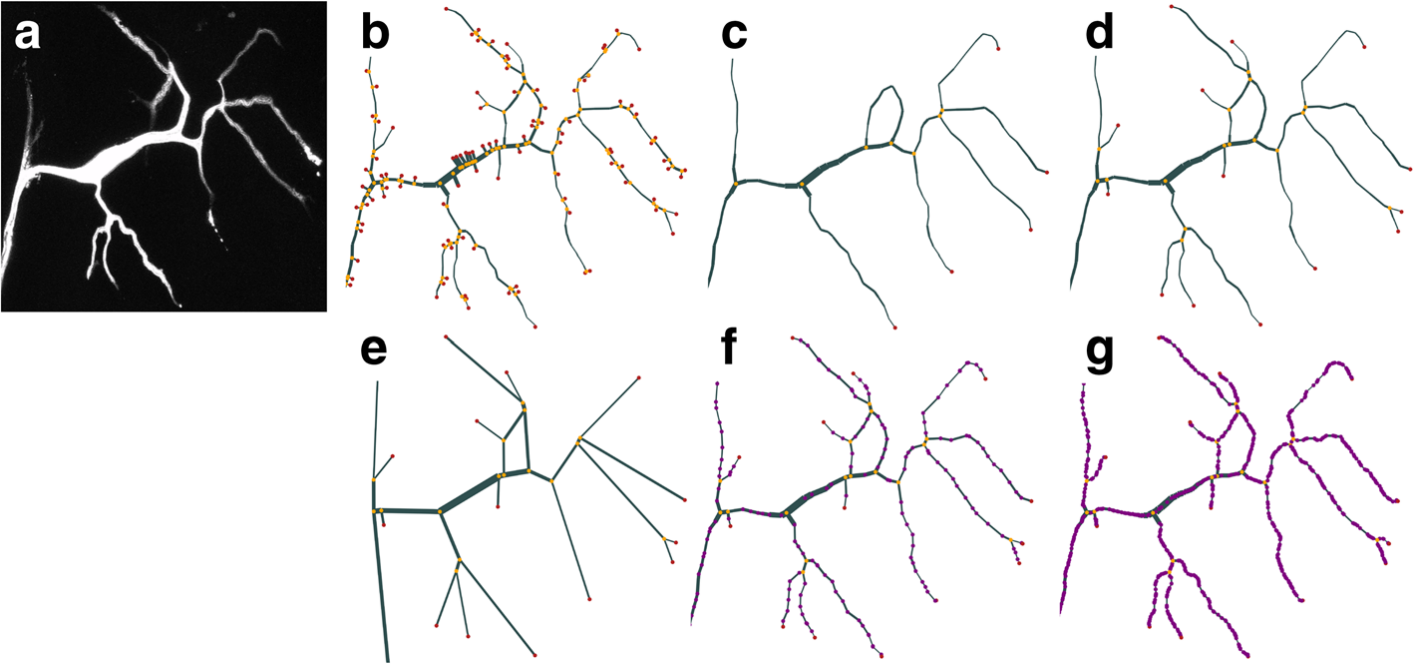
Effects of the pruning and redundancy parameters on the vectorized graph. The *colors yellow*, *red* and *purple* indicate junction, tip and redundant nodes respectively. In the plots, edge widths have been downscaled by a factor of 0.5 to improve plot clarity. **a** Detail of the image *tracheole2* from which the networks were extracted. **b** No pruning (*p*=0), surplus branches are visible. **c** Too much pruning (*p*=50), information is lost. **d** Adequate choice of *p*=3, surplus branches are gone, genuine information is preserved. **e** Only junctions and tips are preserved - poor approximation of the geometry (*r*=0). **f** Half of the redundant nodes are preserved: fair approximation of the geometry (*r*=1). **g** All redundant nodes are preserved - good approximation of the geometry (*r*=2)

The pruning option has to be handled with great care. Depending on the type of network, small branches could be a major source of information or they could be completely irrelevant. We advise to only prune away branches that are significantly shorter than the average branch length, thus ensuring that most of the removed branches are artifacts and not genuine features of the network. Figure 5 b to d show the effect of no pruning, too much pruning and correct choice of *p* (in this case *p*=3) on the network extracted from the original image (detail of *tracheole2*) shown in Fig. 5 a.

**Redundancy** **-r** Setting the redundancy parameter *r*∈{0,1,2} does not change the behavior of the network extraction mechanism but influences in how much detail the final extracted network is saved to the hard drive. Initially, the extracted network contains many points that only support the geometry of the network and have no significance for the topology. These points do not represent any junctions or endpoints and we therefore call them *redundant* points.

- Removes all redundant points in the final network representation. This reduces the size and complexity of the resulting data structure significantly and is handy if the geometry of the network is not important or the network is very large.
- Removes half of the redundant points and therefore is a good way to reduce the size of the data structure but still have an acceptable approximation of the network’s geometry.
- All redundant points will be saved. This is the best option if parameters like the angle at junctions or curvature of edges need to be measured as accurate as possible.

Keeping more redundant points is always a tradeoff between size and speed on one hand and an accurate description of the network’s geometry on the other hand. Figure 5 e to g show the final network with no, half and all redundant points respectively. By default redundancy is set to zero and only a graph with no redundant nodes is saved.

### Step 3: network manipulation

So far *NET* only works for two-dimensional images. In theory our approach is not constrained to two dimensions. However, segmentation of three-dimensional images poses several challenges which we were not able to overcome so far.

*NET* was designed for planar networks - graphs that can be drawn without any edges crossing. The examples used in this publication include projections of *Drosophila* tracheoles on a plane where in reality these structures grow in three dimensions. However the tracheoles follow internal surfaces of the insect [25, 26], therefore the two-dimensional projection is very close to the structure itself and the extracted graph can be assumed to be a faithful representation of the depicted network. Nevertheless, projection introduces systematic error such as a distortion of edge lengths as well as potential edge crossings, which will result in spurious nodes. In order to remove the spurious nodes, we have to give up full automation of the process, as it is very challenging to create an algorithm that reliably distinguishes between real and false junctions in the projection. However, the number of spurious junctions, although typically non-zero, is limited. Manual correction of artifacts in such cases is possible and warranted. To make the process of spurious junction elimination and correction of artifacts in the graph fast and easy we have created a graphical user interface for graph manipulation - the *GeGUI*.

**Fig. 6:**
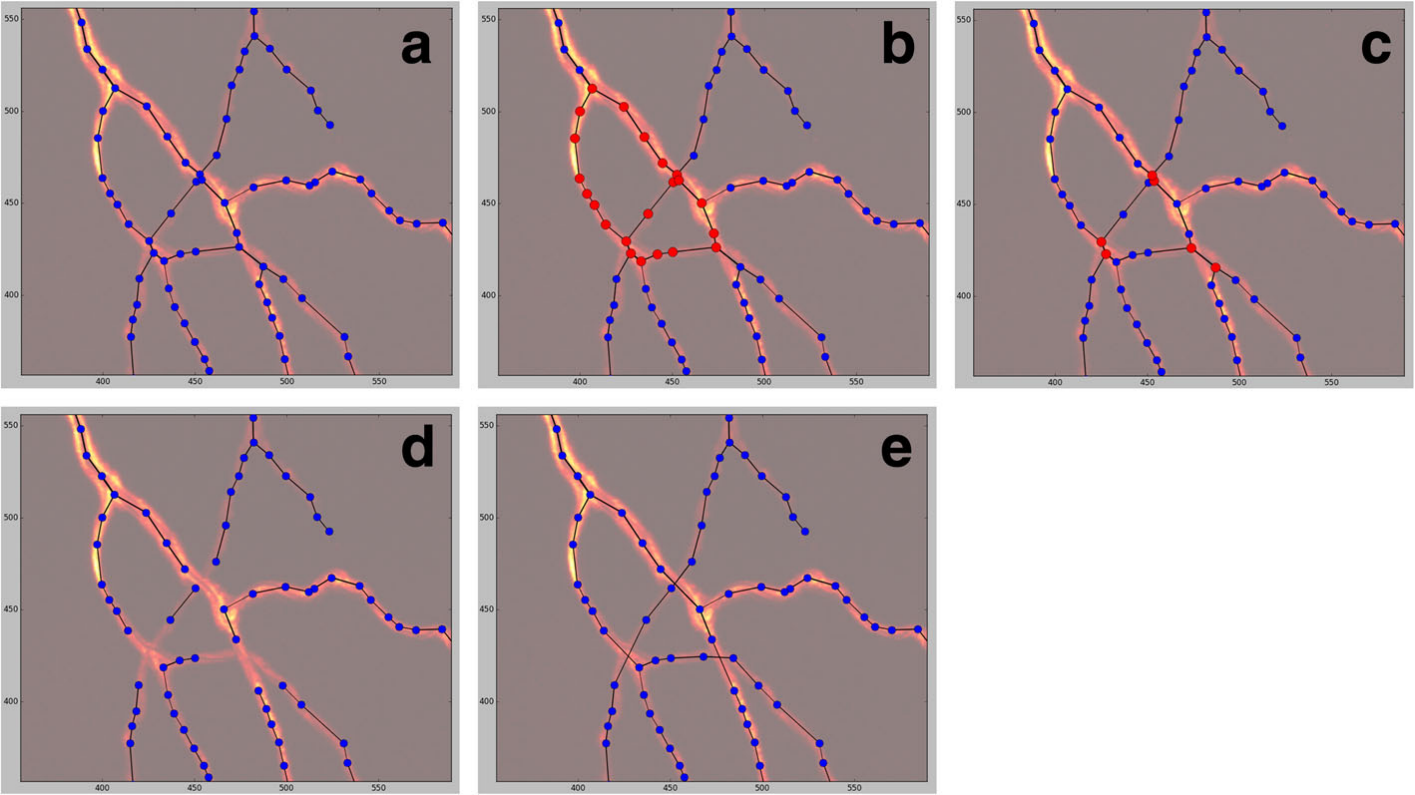
Illustration of spurious node elimination with the *GeGUI*. **a** Detail of the extracted graph superimposed onto a detail of the original microscopy image *tracheole1* (image in false-colors to improve contrast). **b** Highlighting of all loops still present in the graph to facilitate elimination of spurious junctions. **c** The nodes that form spurious junctions can be selected individually by clicking. **d** Deletion of selected nodes. **e** The final version of the graph with all spurious junctions corrected and no cycles left

After the network has been successfully extracted and saved, it can be displayed and manipulated using the GUI. It will load the extracted network and superimpose it on the original image. This is done to facilitate work for the human operator: By having the original image directly beside the extracted network, it is easier to recognize where mistakes were made and what needs to be done so the extracted network closer resembles the real structure. To facilitate usage, when creating a new node the radius at the position of the new node is automatically measured. An illustration of the rewiring of spurious junctions using *GeGUI* is shown in Fig. 6 a to e.

The *GeGUI* needs three files to correctly operate: the extracted network, the original image and the distance map of the image created during the extraction process (the user can simply enable -dm while running *NET* to save the distance map). To run *GeGUI* with a given network file, the user can provide the script with the path to a folder were all these three files are located for a given network:



In the data/results folder of the repository the user can find the three folders tracheole1, tracheole2 and tracheole3 containing the necessary files resulting from the processing of the example images. To load the graph extracted from *tracheole1*, the user can run:



Using the GUI involves point-and-click commands to mark and create nodes as well as key-press commands to delete nodes, create edges and switch between options. After work on the graph is completed, the new graph will be saved as *.gpickle* file and can then be either reloaded and further edited or used for measurements.

### Step 4: network statistics

We provide a script, analyze.py to quantify some select basic properties of any network created either directly by *NET* or manipulated with *GeGUI*. The script measures quantities like number of nodes or total length of the graph and saves them to a text-file. To analyze the properties of the network from *tracheole1*, the user can run:



If more complex or combined measurements are needed, the script can be expanded or adapted quite easily as it is a modular collection of measurement functions.

## Results

### Validation

To assess the quality of networks extracted with *NET*, we re-extracted known networks from 389 images of *Drosophila* tracheoles and then compared them to the original graphs. We created an artificially noisy background, using spatially correlated noise, and plotted the known networks on this background with varying intensity for the edges to make them more similar to real world images. Then we segmented these artificially created images again and extracted networks to compare them with the original networks. The statistics we use for the comparison aim to capture all important aspects of a network’s topology and geometry: The total number of nodes *N* in the graph hints, whether the topology has been correctly resolved. To validate the network’s geometry, we compare the total length *L* i.e. the sum over individual edge lengths of the networks. Moreover we compare the average edge weight $\bar {R}$ and the ratio of the biggest to the smallest edge weight *r*=min(*R*)/max(*R*). For each observable, the error *σ* made during extraction is quantified as

$$ \sigma_{S} = \frac{\left|S_{o} - S_{v}\right|}{S_{o}} $$

where *S*_*o*_ is the respective observable measured in the original network and *S*_*v*_ the observable measured in the artificially created validation network. All values for *σ* shown in Table 1 are the mean over all 389 extracted and re-extracted networks. The above-mentioned statistics leave the comparison invariant under node translations and squeezing or stretching transformations. To rule out that such a transformation has occurred, we need to assess whether the two networks *look* the same in real space. Therefore we plotted both networks without any translation or fitting to increase overlap and calculated the sum over the pixel-wise difference between both images normalized by the total amount of pixels in the original network. We did this for several dpi values for the plots to exclude influences of plot resolution on the results. Values given in the table have been calculated for a resolution of 80 dpi. The comparison of known to extracted networks quantifies the error that is introduced during one pass through *NET*. *σ*_*N*_ and *σ*_*L*_ lie in acceptable ranges of not more than 10% deviation from the original network whereas the edge weights in the re-extracted networks deviate by about 25%. This can be explained as a systematic error stemming from image preprocessing techniques like blurring and binary opening and closing that affect edge weights far stronger than edge lengths. The average pixel-wise difference between original and re-extracted network is very small, giving proof that the network’s geometry is resolved very well and *NET* does not introduce any artificial transformation to node positions.

**Table 1:** Comparison of node number *N*, network length *L*, mean edge weight $\bar {R}$, ratio of smallest to largest weight *r*=min(*R*)/max(*R*) and pixel-wise difference *D* of the plotted networks

*σ*_*N*_	*σ*_*L*_	$\sigma _{\bar {R}}$	*σ*_*r*_	*D*
9.0±8.6*%*	2.6±4.6*%*	25.6±10.0*%*	24.1±20.1*%*	0.1±0.1*%*

For each value, the respective observable was calculated in the known and the re-extracted network and the absolute difference normalized by the value in the known network was calculated. The errors *σ*_*N*_, *σ*_*L*_, $\sigma _{\bar {R}}$, *σ*_*r*_ and *D* shown in the table are the mean error and its standard deviation for 389 automatically extracted and re-extracted networks by *NET* from images of *Drosophila* tracheoles. Error distributions are heavily skewed towards small values, errors cannot be negative

We uploaded all images used for the validation as well as all the statistics calculated for the networks to the validation folder in the repository. The full validation process is described in more detail in the Additional file 1 and can be reproduced by the reader by running the validation.sh script. Users that heavily rely on the edge weight extracted by *NET* need to be wary of the error in $\bar {R}$ and *r*, as it is substantial. If edge weights are of major importance, carefully adapting the parameters used for segmenting the images to potentially reduce the errors should be considered. Furthermore running the validation.sh script plots histograms of all error distributions. This can be used to make an informed decision on the errors and their impact and meaning for the specific dataset.

### Processing speed

Most of the algorithms of *NET* have been ported to C using cython [27], therefore the script is able to handle extremely large networks with millions of nodes. This has already been taken advantage of in the extraction and analysis of leaf venation patterns [19].

To test how long different kinds of networks take to be processed with our framework, in Table 2 we list processing times for networks with a number of nodes (including redundant nodes) in the range of 10^2^−10^6^. Network extraction time scales linearly with number of nodes and loading and writing times for images can vary depending on file format. The processing times were measured on an Intel Core i7-3770 CPU @ 3.40 GHz x 8, 31.4 GB Memory, using one of the eight kernels of the CPUs. Visualization of the graphs was disabled for these measurements and all graphs were saved with all redundant nodes. Even for graphs with 10^6^ nodes, the processing time was under 5 min.

**Table 2:** Processing speed for different types of networks with node numbers in the range 10^2^−10^6^

Image	*N*	Time [ *s*]
*Tracheole7*	1.9·10^3^	2.5·10^−1^
*Bubbles1*	4.7·10^3^	1.6·10^0^
*Tracheole3*	6.1·10^3^	6.6·10^−1^
*Cracks2*	8.7·10^4^	1.3·10^1^
*Leaf2*	1.3·10^5^	2.7·10^1^
*Leaf3*	1.1·10^6^	1.8·10^2^

Processing speed appears to scale linearly with number of nodes as soon as I/O overhead can be neglected in comparison to the time spent executing the processing algorithms. All graphs were processed with parameters *p*=3, *r*=2, *v*=*T* *r* *u* *e*

## Discussion and conclusions

Although *NET* offers some functionality with regards to image preprocessing and segmentation, its main strength lies in the extraction of a graph from an already segmented image. Its ability to extract nodes with a degree of two - nodes lying on a line and not an intersection - enables it to capture the geometry of a network with curved edges. With regards to speed and accuracy, *NET* performs on a comparable level to other network extraction tools and yields graphs that closely approximate the networks depicted in the images. *NET* also extracts edge weights based on the diameter of the edges in the image. Although the edge weights extracted by *NET* are dependent on image preprocessing and segmentation of the input image, its ability to extract edge weights at all is rare among comparable network extraction tools. A possibility to further improve *NET*’s speed is the parallelization of graph creation from the triangulation. For large networks this is the process that is most time-consuming. We did not explicitly incorporate multi-threading into *NET* as the framework is designed with batch-processing in mind and can therefore simply be run multiple times on different portions of the dataset to use all available CPU resources and increase efficiency.

In this publication we mostly show example applications from biology but the usage of the framework does not need to be limited to this kind of networks. It can be used in a broad array of datasets to detect and measure elongated shapes. Moreover every boundary of a structure can be represented by a network-like structure by using edge detection like the Sobel filter on the image. The image of the bubbles we use as an example can be created this way by using the script binarize_bubbles.py This has a wide range of applications, such as the extraction of crack patterns in dried clay or the tracking of bubbles in microfluidics. Several examples of the networks extracted by *NET* - biological or not - alongside the original images are shown in Fig. 1 a to f.

Nevertheless we created *NET* mostly with application in the life sciences in mind. In our experience, manual processing is prevalent in these laboratories. Our close collaboration with the Leptin Lab at EMBL Heidelberg and Douglas Daly at the New York Botanical Garden has enabled us to simplify workflows for the scientists working with *NET* and switch to automated processing. Apart from saving time, automated processing also has the advantage of yielding reproducible results and avoiding human error: given a binary image and set of processing options, *NET* will always yield the same results, whereas manual measurement largely depends on the perception of the individual performing the measurement. Automated processing also enables us to measure more complex quantities like the area of the convex hull, curvature and angles, or other topological metrics of the network. More importantly it also works for extremely large networks where manually measuring metrics across the whole network is simply not feasible.

Last but not least we are actively developing and maintaining *NET*. In the future one can expect to see the incorporation of some more sophisticated segmentation algorithms into the framework as well as a port to python 3. Nevertheless we are aware that we cannot compete with the most advanced segmentation toolkits available as our focus is more on the networks-part of the software. We plan to expand *GeGui* as it has proven to be a very useful tool in our daily work with graphs. In the future we want to include more functionality like improved drawing of graphs from scratch as well as the ability to deal with disconnected components and multiple graphs at the same time. Furthermore we want to improve *NET*’s accuracy with regards to the extracted edge weights as we feel this is the only aspect of *NET*’s performance that is still lacking. We want to emphasize that both the source code for the network extraction as well as for the manual graph handling can be found in the Git repository [10]. We expect that these tools will prove especially useful in facilitating quantitative analysis of large datasets. We are very happy if our software is used and we are open to suggestions regarding improvement of existing functionality, additional features or fixing of bugs. To communicate with us, we encourage the reader to use the issue-tracker system provided by GitHub.

## Availability and requirements

**Name:** Network_extraction**Home page:** https://github.com/JanaLasser/network_extraction **Operating systems:** Linux, Mac, Windows**Programming language:** Python**Licence:** Copyright (C) 2015 Jana Lasser Max Planck Institute for Dynamics and Self Organization Goettingen **This program is free software:** you can redistribute it and/or modify it under the terms of the GNU General Public License as published by the Free Software Foundation, either version 3 of the License, or (at your option) any later version. This program is distributed in the hope that it will be useful, but WITHOUT ANY WARRANTY; without even the implied warranty of MERCHANTABILITY or FITNESS FOR A PARTICULAR PURPOSE. See the GNU General Public License for more details. You should have received a copy of the GNU General Public License along with this program. If not, see http://www.gnu.org/licenses/.

## Additional file

Additional file 1 Supplement. (PDF 178 kb)
