# Supplementary material for: NET: a new framework for the vectorization and examination of network data
Source: Source Code Biol Med. 2017 Feb 8;12:4. doi: 10.1186/s13029-017-0064-3 (PMC5299731; doi:10.1186/s13029-017-0064-3)
Supplement: Additional file 1 — Supplement. (PDF 178 kb) [file 13029_2017_64_MOESM1_ESM.pdf]

Supplement

The first part of the supplement contains a complete list of the options available for the scripts used to segment and vectorize images and handle and analyze graphs as well as additional usage examples. The second part gives a more in-detail and technical description of the algorithms used by *NET* to vectorize images. In the third part we detail how the validation of *NET*'s results is performed.

S1 Processing scripts

Segmentation, vectorization, graph manipulation, validation and analysis are handled by one script each, stored in the folders `/binarize`, `/net`, `/gegui`, `/validation` and `/analyze`.

S1.1 Segmentation

For segmentation of images we use a combination of blurring, adaptive thresholding and morphological operations on the binary image. The processing steps to generate a binary image from a dataset using `binarize_adaptive.py` images are:

- Gaussian blur to reduce small frequency noise.
- Creation of a binary image using adaptive thresholding.
- Removal of small foreground components.
- Binary opening and closing to smoothen the contours.
- Removal of small foreground components.
- Filling of small holes.

The script supports the following options to modify processing parameters:

- -g Kernel size for the Gaussian blur applied to reduce noise in the image.
- -s Kernel size for the binary opening and closing operations applied to smooth the contours of the features.
- -t Neighborhood size for the adaptive thresholding.
- -c Constant specifying that every intensity value below the constant have to be labeled as background.
- -m Maximum size (in pixels) of features that will be removed during the cleanup process.
- -i Flag to invert image if foreground happens to be dark on a light background.

All images shown in this publication were segmented with this script, the parameters used for the segmentation can be found in the file `binarize_adaptive-parameters.txt`.

The only exception to this is the `bubbles1.png` image as its segmentation involves edge detection rather than foreground/background separation, therefore we separated the segmentation process into a different script called `binarize_bubbles.py`. To segment for example the image `cracks1.png`, the user can run

```
python binarize_adaptive.py
../data/originals/cracks1.png -g 3 -s 0 -t 71 -m 7000 -i
```

S1.2 The *NET* script

The complete source code is available at [2]. The main script used for extracting network data is the `net.py` script which relies on the libraries `net_helpers.py` and `C_vectorize_functions.so` which provide helper functions and a cythonized version of the computationally expensive processing steps respectively.

The script is run via the command-line. The only required argument is the path to the binary image that will be processed. Optional arguments are:

- `-dest` directory at which the results will be saved if different from source directory (default `dest` = source directory).
- `-v` Enables program verbosity (default `v` = False).
- `-d` Enables debugging which will increase program verbosity even further and create additional plots of the extracted contours, the triangulation and an overlay of contour, triangulation and extracted graph (default `d` = False).
- `-plt` Enables plotting of the extracted graph (default `plt` = False).
- `-n` Controls the size of the nodes displayed in the plot (default `n` = 4).
- `-fformat` Specifies the format the plots will be saved in (default `fformat` = *.pdf*).
- `-gformat` Specifies the format the graph will be saved in (default `gformat` = *.gpickle*)
- `-dpi` Specifies the resolution of the plots (default `dpi` = 500).
- `-p` Length of surplus branches which will be cropped (default `p` = 5).
- `-r` Level of redundant nodes preserved in the final graph (default `r` = 0). Choices are:
  - 2 all redundant nodes will be preserved
  - 1 roughly half of the redundant nodes will be preserved
  - 0 all redundant nodes will be removed

- `-m` If the binary image has not been cleaned up during the binarization process, artifacts can be removed with this parameter. Every feature smaller than minimum feature size (in pixels) will be discarded (default `m` = 3000).
- `-s` If smoothing has not been performed in the binarization process, it can be enabled with this parameter which also controls the kernel size of the morphological operators applied (default `s` = 0).

Verbosity `-v` and Debug `-d` By default, the script will not produce any command line text output to avoid spam. As processing times are sometimes long and it can be helpful to know the current status of the script, running the script with `-v` enables verbosity. In verbose mode the script will print a notification for each major processing step to the command line as well as the time it took to complete this step.

If something goes wrong and the output of the network extraction is not the desired result, it may be helpful to enable the debug option. This will print even more information about the state of the script to the command line. It will also save snapshots of the processed image at major processing steps so the progress of the processing can be evaluated. Saving images to the hard drive is a time-consuming process therefore for normal network extraction we advise to keep debugging turned off.

Plotting `-plt`, figure format `-fformat` and resolution `-dpi` By default, the script will only create the data-file representing the network. If visualization is needed, plotting can be enabled via `-plt`. This will create a plot of the network where nodes are represented as dots and edges are represented as lines connecting the dots. By default, the created plot will be saved as *.pdf.* A different format can be chosen via the `-fformat` option. For large networks, plotting can take a very long time, especially if the format is *.pdf.* If visualization is needed nevertheless, a way to reduce plotting time is to save plots as *.png* or *.jpg.* The resolution of these plots can be controlled by specifying the `-dpi` parameter.

Graph format `-gformat` and Distance Map `-dm` If the user intends to use the extracted graphs in another program, we recommend saving graphs in a python-independent format like *.gml.* The graph format can be changed by specifying

the `gformat` parameter. A complete list of available formats can be found at networkx's [3] documentation concerning reading and writing graphs.

If the user intends to display and manipulate graphs using *GeGUI*, a distance map of the binary image will be needed. The distance map is needed during the graph extraction process. To save it during a pass of *NET*, simply enable the *dm* switch. To extract a graph from a binary image with $r = 1$, $p = 2$, $plt = True$, $gformat = gml$, $dm = True$ and $v = True$, the user can run

```
python net.py
/dir/subdir/binary_image.png -r 1 -p 2 -plt -gformat gml -dm -v
```

This will create a network and prune away spurious branches shorter than two nodes. It will save the graph as *.gml* retaining half of the redundant nodes needed to support the geometry. Additionally it will create a visualization of the graph and safe it as *.pdf*. The distance map created during the processing will be saved at the location of the resulting graph. Because verbosity is enabled the script will notify the user about its progress with command-line output.

S1.3 The *GeGUI* script

GeGUI combines manipulation of graph objects from `networkx` with `matplotlib`'s plotting capabilities and provides point-and-click functionality for the creation and removal of nodes and edges in the graph. Furthermore the GUI makes use of human capabilities to segment images by superimposing the graph onto the original unprocessed image of the network (not the binary version). This way, for each junction the operator can see the original surroundings of the node in question and manipulate the graph accordingly. For a newly created node, the script measures the radius of the network in the original image at the position of the node and then assigns this radius as weight. Newly created edges will have their weight set as an average of the weights of the nodes they connect.

To further facilitate detection of spurious junctions we make use of the additional information that *Drosophila* tracheoles do not form loops. Therefore every cycle still present in the graph has to originate from at least one false junction. The GUI provides a functionality to highlight all remaining cycles in the graph. The steps to correct false junctions are:

- Mark the nodes which are part of a spurious junction by clicking on them.

- Delete marked nodes (key press “d”).
- Mark each two nodes that are now disconnected but should be connected in the genuine topology by clicking on them.
- Create an edge between the marked nodes by pressing “e”.
- Repeat until all spurious junctions are rewired.

All the source code is available at [2]. The main script used for graph manipulation is `gegui.py`. It relies on three sub-classes to handle user-interaction (`InterActor.py`), manipulation of the underlying graph (`graphHandler.py`) and plotting (`PlotHandler.py`).

The script is run via the command-line:

```
python gegui.py /resultfolder
```

As argument it needs the path to a directory in which the graph, the original image and the distance map of the binary image are located:

```
/resultfolder
-- graph.gpickle
-- dm.png
-- original_image.png
```

Once the script is called it will display a standard `matplotlib` [4] interactive plot showing the graph superimposed onto the original microscopy image. The interactive window supports zooming, taking a screenshot and moving the scope around. We have several options to proceed by entering different keywords on the command-line:

- `m` Enables the “manipulation mode” which provides functionality for the manipulation of the graph explained below
- `x` Exits the script.
- `h` Shows a help-message explaining the options.
- `digraph` Creates a directed graph from the existing graph. For this option to work, exactly one node needs to be selected which will act as the root of the graph.

The command line is not directly needed if the user has entered the manipulation mode. All further clicks and key presses will be performed with the plot in the foreground. Possible actions when in manipulation mode are:

`click` When clicking near a node it will be marked, when clicking near the same node again it will be unmarked.

When shift-clicking on the plot a new node will be created at the position of the click.

`a` Clears the current selection of nodes.

`m` Highlights all remaining cycles in the graph.

`d` Deletes all currently selecting nodes.

`e` Creates an edge between two selected nodes (will fail if not exactly two nodes are selected).

`z` Undoes the last action.

The *Analyze* Script

Running the `analyze` script on a graph will create the text file `network_statistics.txt` in the directory of the graph. The statistics calculated and printed in the text file are

- **Number of junctions**: Every node that has more than two neighbors qualifies as junction.
- **Number of endpoints**: Every node that has only one neighbor qualifies as endpoint.
- **Length of the network**: Sum over the lengths of all edges.
- **Average edge length**: Average length of edges in the network.
- **Average edge radius**: Average radius of edges in the network.
- **Area of the network**: Sum over the area occupied by all edges (edge length times edge radius).
- **Area of the convex hull**: Area the convex hull of the network occupies.
- **Number of cycles**: Number of cycles (closed paths) the network contains.

To generate statistics from a graph, run

```
python analyze.py /dir/subdir/graph.gpickle
```

S2 NET technical details

The algorithms described in the following were all implemented in python. The source-code as well as the fully functional processing script is available online at [2]. In the following we will describe the steps and algorithms that allow us to get from

a pixel-representation of the network structure to a vector-based representation without salient information loss.

S2.1 Contour Extraction

The first step is to extract the contours of the network or, more general, the foreground. Each pixel that belongs to the contour is a foreground pixel which has at least one background pixel in its 8-connected neighborhood. We extract the contour using `openCV`'s [5] `findContours`-method which implements the algorithm suggested in [6]. At this point we already perform a first step towards an abstract representation of the network: we discard most of the points belonging to the contour and approximate it by its dominant points. To achieve this we use the Teh-Chin dominant point detection algorithm suggested in [7] which is already incorporated in the contour finding function. This process is illustrated in Fig 1a. At this point the internal network representation is a list of contours where each contour is a list of coordinate-tuples representing the structure's dominant contour points.

S2.2 Triangulation

Following the approach first suggested in [8] and then refined in [9] and [10], we partition the foreground shape by performing a constrained Delaunay triangulation [11]. We use the the segments between every two adjacent contour points as constraints and therefore force them to appear in the triangulation. To execute the triangulation, we utilize the `meshpy` [12] wrapper for python for the `Triangle` library [13] originally written in C. This provides us with the ability to perform triangulations of very complex shapes at C-speed.

To prepare the triangulation process, we first have to identify the outermost contour. We can safely assume that this contour will be the longest and most complex contour and therefore include the most points. We use the contour composed of the most points and insert the other, smaller contours as holes into the largest contour. Before the triangulation we distort the contour points by a small amount of noise ($\approx 10^{-1}$ $[px]$) to stabilize the triangulation algorithm. Triangulation algorithms can fail if they encounter a degeneracy like collinear (i.e. points lie on the same line) or coincidental points and these degenerate cases are not handled automatically. If this can happen, we call the triangulation process numerically unstable. *NET* uses the library `Triangle` [13] to perform the triangulation. As the contour

points are extracted from a pixel representation which is therefore discretized, it is quite possible that sets of collinear points exist. In practice, the triangulation tends to fail for especially large networks. This is likely due to the fact that in a large image the possibility for a degenerate case to appear is very high. Shifting the positions of the points by a small amount makes it highly unlikely for two points to be collinear. After performing the triangulation we again remove the noise by rounding contour points to the nearest integer, so that it does not impact the result of the triangulation.

As illustrated in Fig 1b we can now represent the network by a list of triangles consisting of the coordinate tuples of their vertices. We further follow the approach suggested in [9] and classify the triangles into *junction*, *normal*, *end* and *isolated* triangles based on how many edges they share with the initial contour (i.e. *internal* edges as opposed to *external* edges). As we are only interested in the largest connected component, we discard isolated triangles if there are any left.

S2.3 Skeleton Extraction

Several approaches for the stabilization of singular regions (i.e. branching points) have been suggested [10], [14] to improve the quality of the skeleton. This is done to ensure that the extracted skeleton conforms more to the human perception of the "right" skeleton. Nevertheless in this work, we have developed our own approach to find the best skeleton and improve upon the initial triangulation.

S2.4 Pruning

First we found that especially noisy contours lead to the creation of tiny surplus branches along otherwise straight edges. A common pattern is an end triangle directly attached to a junction triangle or only separated from a junction triangle by a very small number of normal triangles. To improve the outcome of the triangulation, we therefore remove all these very small branches by cropping away all the structures of the form *end* - *normal* - ...- *normal* - *junction* where the number of normal triangles in between most of the times is ≤ 5.

S2.5 Finding the correct triangle center points

Every triangle will contribute one point (a "center" point) to the final skeleton but finding that point for each triangle is not trivial. To improve the accuracy of the approximation of the skeleton we differentiate between the three triangle species when looking for the optimal center point. For end-triangles we simply use the point opposing the single internal edge (i.e. the outermost point) so we do not lose network length at the tips. For normal and junction triangles the process of finding the center point is more involved. The definition of the skeleton is a one pixel thin line which has the same (i.e. maximum) distance from the background. We make use of this definition by creating an Euclidean distance map [15] of the original binary image and then looking for local maxima in this distance map. The distance to the nearest background pixel for each foreground pixel is calculated using the algorithm proposed by [16]. For normal triangles we are looking for a maximum in the distance map along the line bisecting the angle enclosed by the two internal edges. For junction triangles we do the same but look along the longest angle-bisection line.

Looking for the optimal center point in this way has the added benefit of already providing us with the radius of the structure at each center point - we simply have to note the value of the local maximum in the distance map we found.

S2.6 Conversion to a Graph

A graph can be defined by its adjacency matrix which states which nodes are connected to which other nodes. To establish these neighborhood relationships, we first have to create a preliminary triangle adjacency matrix. We do this by iterating over all triangles and looking for edges shared between two triangles which will then be noted as neighbors. Similar to a weighted adjacency matrix we also store the length and radius of each edge as well as the coordinates of each node in the matrix. As the networks processed this way can be extremely large (up to 10^6 nodes for leaf venation patterns) we use sparse matrices provided by the `scipy`-library [17] to store the adjacency matrices. We now use the functionality provided by the `networkx`-library [3] to convert this adjacency matrix into a graph-object.

At this point we can choose to either keep all the nodes we have extracted (Fig 1c) or remove all nodes which are not necessary to depict the topological structure of

the network (Fig 1d). Every node that originates from a normal triangle (i.e. has exactly two neighbors) is a redundant node and only serves to better approximate the geometry of the network.

It has to be mentioned that as we use a triangulation to partition the network, no node in the resulting graph can have a degree > 3. For our purposes it does not matter whether the network at a certain point is represented by two neighboring nodes with degree three or by a single node with degree four. If the degree of the nodes is essential for the analysis one could easily implement a method that combines nodes in close proximity to each other to a single node with a larger degree.

S3 Automated validation

For the automated validation we used the extracted and re-extracted graphs from 389 microscopy images of *Drosophila* tracheoles. The original binary images can be found in the source folder `validation/validation-graphs-tracheoles`. First we extract a graph from the original binary image using the `net.py` script (parameters -p 2 -r 1) and use it as our known graph. The script `create-validation-images.py` creates an artificial image using the known graph and plotting it on top of a noisy background and saves it to the source folder. We then segment this artificial image again using the `binarize_adaptive.py` script (parameters -t 51 -g 3 -s 3 -m 35000 -c 80) and extract a graph from the segmented image (same parameters as for the original). The script `calculate-validation-statistics.py` then calculates the number of nodes, length of the network, mean edge weight, ratio between smallest and largest edge weight and pixel-wise difference between plots of the networks for the original and re-extracted network respectively and saves them to the file `validation-statistics.txt`. The validation statistics calculated this way can then be analyzed by running `analyze-validation-statistics.py` which will calculate the deviation from the known graph for each re-extracted graph, save it to the file `deviations.txt`, plot the distribution of deviations and output a summary of the deviations to the command line. To reproduce the validation conducted in this publication, the reader can run the script `validation.sh` which will perform all the above-mentioned steps on the 390 validation images. Be aware that reproducing the full validation will take a while.

Author details

References

1. Otsu, N.: A threshold selection method from gray-level histograms. IEEE Trans. Sys., Man., Cyber **9**, 62–66 (1979)
2. Lasser, J.: Github Repository for *NET*. \url{https://github.com/JanaLasser/network_extraction}
3. Hagberg, A.A., Schult, D.A., Swart, P.J.: Exploring network structure, dynamics, and function using networkx. In: Proceedings of the 7th Python in Science Conference (SciPy2008) (2004)
4. Hunter, J.D.: Matplotlib: A 2d graphics environment. Computing In Science and Engineering **9**, 90–95 (2007)
5. Bradski, G.: The opencv library. Dr. Dobb's Journal of Software Tools (2000)
6. Suzuki, S., Abe, K.: Topological structural analysis of digitized binary images by border following. CVGIP **30**, 32–46 (1985)
7. Teh, C.H., Chin, R.T.: On the detection of dominant points on digital curve. PAMI **11**, 859–872 (1989)
8. Fan, K.C., Chen, D.F., Wen, M.G.: A new vectorization-based approach to the skeletonization of binary images. In: Proceedings of 3rd International Conference on Document Analysis and Recognition, p. 627 (1995)
9. Zou, J.J., Chang, H.-H., Yan, H.: A new skeletonization algorithm based on constrained delaunay triangulation. In: Proceedings of the Fifth International Symposium on Signal Processing and Its Applications (ISSPA), pp. 927–930 (1999)
10. Zou, J.J., Yan, H.: Skeletonization of ribbon-like shapes based on regularity and singularity analyses. Systems, Man, and Cybernetics, Part B: Cybernetics, IEEE Transactions on **31**, 401–407 (2001)
11. Paul Chew, L.: Constrained delaunay triangulations. Algorithmica **4**, 97–108 (1989)
12. Klöckner, A.: Github Repository for *meshpy*. \url{http://git.tiker.net/meshpy.git}
13. Shewchuk, J.R.: Triangle: Engineering a 2D Quality Mesh Generator and Delaunay Triangulator. In: Lin, M.C., Manocha, D. (eds.) Applied Computational Geometry: Towards Geometric Engineering vol. 1148, pp. 203–222. Springer, ??? (1996)
14. Morrison, P., Zou, J.J.: Achieving a more accurate skeleton through the refinement of skeletonization triangles. In: Proceedings on Digital Image Computing: Techniques and Applications (DICTA), p. 49 (2005)
15. Danielsson, P.E.: Euclidean distance mapping. Computer Graphics and Image Processing **14**, 227–248 (1980)
16. Felzenszwalb, P., Huttenlocher, D.: Distance transforms of sampled functions. Computing and Information Science Technical Reports **14**, 227–248 (1980)
17. Oliphant, T.E.: Python for scientific computing. Computing in Science and Engineering **9**, 10–20 (2007)
18. Schindelin, J., Arganda-Carreras, I., Frise, E., Kaynig, V., Longair, M., Pietzsch, T., Preibisch, S., Rueden, C., Saalfeld, S., Schmid, B., Tinevez, J.-Y., White, D.J., Hartenstein, V., Eliceiri, K., Tomancak, P., Cardona, A.: Fiji: an open-source platform for biological-image analysis. Nature methods **9(7)**, 676–682 (2012)

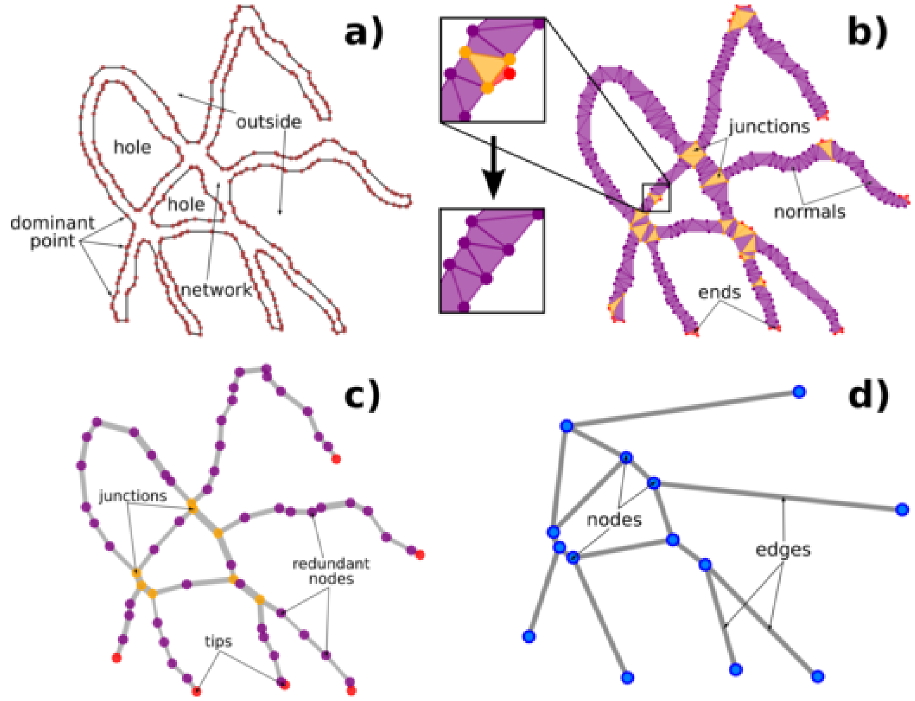

Figure 1: **The skeleton extraction process. (a)**: Dominant points of the contour extracted from the binary image. Structural details of the network are embedded into the outermost contours as holes. **(b)**: Constrained Delaunay triangulation of the contour using the contour segments as constraints. *End*-, *normal*- and *junction* triangles are indicated in red, purple and yellow. The detail shows the alteration of the triangulation by removal of end-triangles directly adjacent to junction-triangles. **(c)**: Graph extracted from the triangulation. There is still a number of redundant nodes originating from normal (purple) triangles left. **(d)**: Symbolic representation of the graph showing only its topology.
